# Development and validation of an instrument to assess the knowledge of general practitioners and pediatricians about photoprotection and solar radiation^☆^^☆☆^

**DOI:** 10.1016/j.abd.2019.09.011

**Published:** 2019-09-30

**Authors:** Fernanda Mendes Araújo, Julliana Andrade do Carmo, Letícia Diniz Cunha, Igor Monteiro Lima Martins, Airton dos Santos Gon, Antônio Prates Caldeira

**Affiliations:** a Department of Clinical Medicine, Dermatology, Universidade Estadual de Montes Claros, Montes Claros, MG, Brazil; b Postgraduate Program in Health Sciences, Universidade Estadual de Montes Claros, Montes Claros, MG, Brazil; c School of Medicine, Faculdades Integradas Pitágoras de Montes Claros, Montes Claros, MG, Brazil; d Department of Clinical Medicine, Dermatology, Universidade Estadual de Londrina, Londrina, PR, Brazil; e Department of Pediatrics, Universidade Estadual de Montes Claros, Montes Claros, MG, Brazil

**Keywords:** Health education, Skin neoplasms, Solar radiation, Sunscreening agents, Ultraviolet rays, Validation studies

## Abstract

**Background:**

The knowledge of general practitioners about photoprotection is unknown.

**Objectives:**

To develop and validate an instrument to evaluate the knowledge of general practitioners and pediatricians about photoprotection, gauging the knowledge of these professionals.

**Methods:**

The study followed the steps: (1) Literature identification and item elaboration related to the theme; (2) Content validation; (3) Apparent validation; (4) Construct validation: internal consistency analysis and discriminatory analysis; (5) Reliability analysis. In Step 4, the instrument was applied to 217 general practitioners and pediatricians who worked in the host city of the study; the scores were compared with dermatologists scores.

**Results:**

The final instrument had 41 items and showed satisfactory internal consistency (Cronbach's alpha = 0.780), satisfactory reproducibility and good test–retest reliability (good-to-excellent kappa statistic in more than 60% of items). The discriminatory analysis registered a mean score of 54.1 points for dermatologists and 31.1 points for generalists and pediatricians, from a total of 82 possible points, representing a statistically significant difference (*p* < 0.001). Generalists and pediatricians demonstrated an understanding of the relationship between excessive sun exposure and skin cancer, but they revealed lack of technical information necessary for their professional practice.

**Study limitations:**

The instrument evaluates only knowledge, without evaluating the conduct of the participants.

**Conclusion:**

The results show that the instrument has good internal consistency and good reproducibility. It could be useful in the identification of general practitioners and pediatricians knowledge gaps on the subject, for the subsequent development of training and educational strategies.

## Introduction

Skin cancer is the most prevalent neoplasm in Brazil and the world; approximately two million new cases were registered in the United States alone, representing a significant burden for that country health system.^1^, ^2^, ^3^ Skin exposure to ultraviolet (UV) radiation, resulting from prolonged and unprotected sun exposure, is the main environmental risk factor associated with the onset of skin cancer.^4^, ^5^, ^6^ The way sun exposure occurs is a determinant in the affected cell line: chronic and cumulative exposure is associated with the onset of squamous cell carcinoma, whereas acute and intermittent exposure is more associated with basal cell carcinoma and melanoma.^7^

To date, exposure to UV radiation is the only established modifiable cause of melanoma; thus, primary prevention strategies for skin neoplasms focus on limiting UV exposure through sun-protective behaviors.^8^, ^9^ Effective sun protection includes the adoption of several measures, including environmental, mechanical, topical, and systemic photoprotection, as well as photoprotection education.^10^, ^11^

Of all preventive measures, photoprotection education is perhaps the slowest in reducing the incidence of skin cancer, but it is certainly the largest and most effective investment in population health, especially the pediatric population.^12^ When compared with the high cost of treatment of skin neoplasms, effective implementation of preventive measures can lead to a significant reduction in the resources used by health systems.^6^

Statistics from the Brazilian Society of Dermatology (Sociedade Brasileira de Dermatologia [SBD]) show that, among the Brazilian population, sun protection is far from adequate.^13^ Educational campaigns in Australia have increased the adoption of protective measures at leisure times from 12% to 48% over a ten-year period.^14^, ^15^ However, although the population of that country is more aware of the importance of photoprotection in the prevention of skin cancer, adherence to photoprotection practices remains suboptimal; less than half the population adopt sun protection measures during outdoor activities.^14^, ^15^ Olsen et al. observed that the educational level was strongly associated with the use of photoprotectors and, to a lesser extent, the use of hats; furthermore, these authors indicated that adherence to the use of photoprotectors was higher in women than in men.^14^

The most successful programs in photoprotection education are those that contemplate diverse and complementary approaches; the objective is that the knowledge leads to a significant change of attitudes and behaviors in the population.^12^ Photoprotection guidelines are part of the routine of the dermatologist.^11^ However, general practitioners and pediatricians have a paramount role in the prevention of skin cancer, since they represent physicians with great educative power.

In Brazil, the increasing expansion of primary care teams observed in recent years reinforces the role of general practitioners and family/community doctors in promoting educational activities, including the promotion of effective photoprotection. The World Health Organization (WHO) advocates that photoprotection programs are urgently needed to promote greater awareness of UV radiation damage and to foster changes in lifestyle habits associated with increased exposure and risk for skin cancers.^16^ The American task force for the reduction of skin cancer has strongly recommended that children, adolescents, and young adults be advised on the appropriate photoprotection measures, given the importance of early exposure to UV radiation.^17^

Despite the context presented, little is known about the protection guidelines provided by general practitioners and pediatricians. Moreover, little is known about the knowledge of healthcare professionals about photoprotection measures.^18^ Knowing the theoretical background of these professionals with regard to photoprotection is of vital importance in the strategic planning of educational activities on the subject. No Brazilian studies evaluating the knowledge of general practitioners and pediatricians on the subject using validated instruments were retrieved from the literature. The present study aimed to develop and validate an instrument to assess the knowledge of physicians on photoprotection.

## Methods

### Study design and ethical aspects

This was a cross-sectional study in which an instrument to assess the knowledge of general practitioners and pediatricians about photoprotection and solar radiation was elaborated and validated. The project of this study was approved by the Ethics Committee of the present institution (opinion No. 1,792,189). The research objectives were explained to all participants, who subsequently signed an informed consent form.

**Figure 1:**
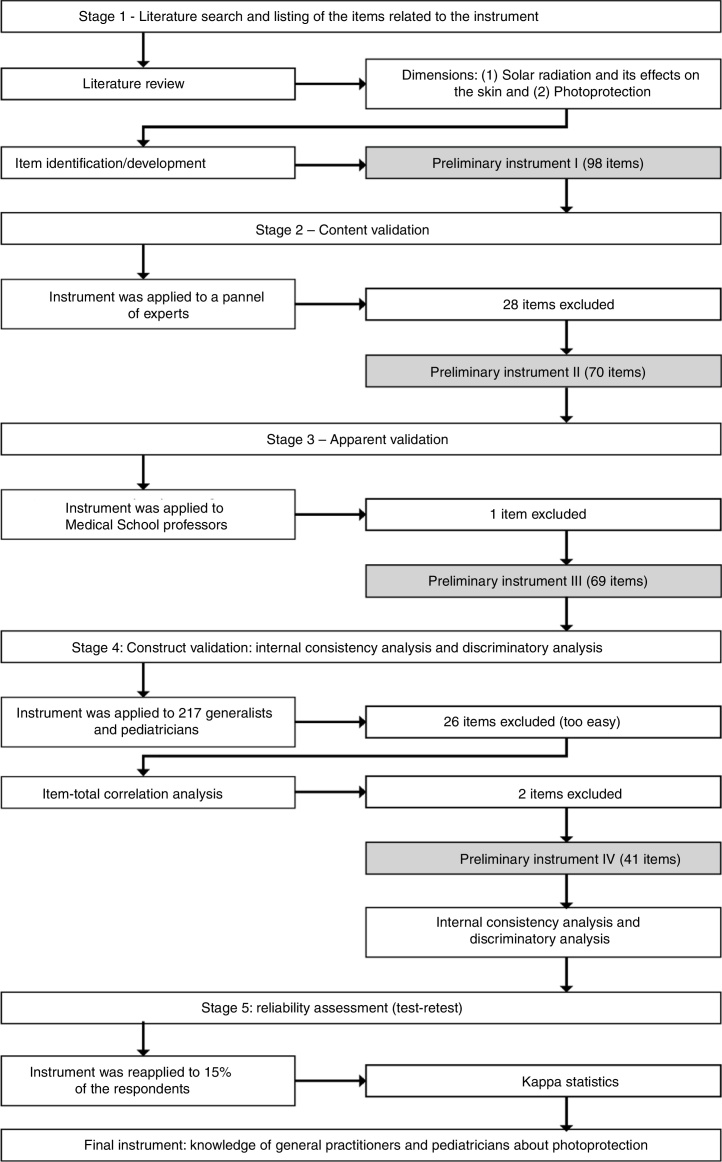
Summary of the steps for the development of the instrument “The knowledge of general practitioners and pediatricians about photoprotection and solar radiation”.

The study followed the following steps: (1) Identification of the relevant literature and listing of the items related to the instrument; (2) Content validation; (3) Apparent validation; (4) Construct validation, with internal consistency analysis and discriminatory analysis (or hypothesis test); (5) Reliability analysis, as shown in Fig. 1.

#### Step 1: Identification of the relevant literature and listing of the items related to the instrument

For the identification of the basic literature for the listing of the items, a search was conducted in the Medical Literature Analysis and Retrieval System Online (MEDLINE), the US National Library of Medicine (PubMed), and the Scientific Electronic Library Online (SciELO) databases, using the terms solar radiation, sunburn, photoaging of the skin, photoprotection, and sun protection factor, and their respective equivalent terms in Portuguese. In addition to scientific articles, the research also sought to identify protocols, guidelines, and instructional materials directed to healthcare professionals. The search was conducted between April and May 2015. Texts not entirely in Portuguese, English, Spanish, or French were excluded. After the retrieved material was assessed, the references for the listing of the items were defined.^8^, ^11^, ^13^, ^16^, ^17^, ^19^, ^20^, ^21^, ^22^, ^23^, ^24^ The synthesis of the main contents resulted in 98 items, which were transformed into short and objective statements in order to create the instrument to be validated, encompassing two different dimensions: (1) Solar radiation and its adverse effects on the skin; and (2) Photoprotection.

#### Step 2: Content validation

The instrument was submitted to analysis of the content and the semantic structure by four specialists in the field (dermatologists from different regions of the country), who evaluated the relevance of each item (irrelevant, somewhat relevant, relevant, or very relevant) and clarity and appropriateness of the assertion (clear and adequate or inadequate). After the analysis by the experts, the instrument was reformulated; items considered clear, adequate, and relevant or very relevant by at least three of the evaluators were maintained. Some items had their writing adapted, as suggested by the evaluators, and then 70% of the items were maintained as true statements, similar to the reference text, and 30% were randomly transformed into false assertions.

#### Step 3: Apparent validation

After the evaluation of the specialists, the new instrument was then applied to six professors of undergraduate medical courses that teach disciplines related to clinical medicine and pediatrics, who answered the questionnaire and evaluated the clarity and comprehension of each item.

#### Step 4: Construct validation: internal consistency analysis and discriminatory analysis

To identify the physicians, the authors requested lists with a nominal relation of generalists and pediatricians who worked in health and educational institutions in the study host city, including the public and private systems, outpatient clinics, and hospital services. There was no sample calculation. All 298 physicians identified were personally approached at their workplace and asked to answer the questionnaire. Three attempts were made to approach each physician, with a weekly interval between each approach; 221 physicians agreed to participate. Data collection occurred during a four-month period from December 2015 to March 2016. During data processing, four questionnaires were excluded because they had been applied to physicians of other specialities other than the two considered in this study. Thus, 217 physicians answered the instrument at this stage, which also included items to characterize the respondent. Each statement was followed by five Likert scale response options (“I fully agree,” “I partially agree,” “I do not agree or disagree,” “I partially disagree,” and “I strongly disagree”). Professionals were asked to indicate their level of agreement or disagreement regarding the affirmative, according to the level of knowledge on the subject, and could also indicate that they had no level of agreement or disagreement (“I do not agree or disagree”), assuming ignorance about the item. Then, the level of correlation between the items of the instrument was assessed, as well as the internal consistency of the instrument through Cronbach's alpha.

For discriminatory analysis or hypothesis testing, the instrument was applied to 20 dermatologists working in the host city of the study. The answers of the generalists and pediatricians, and those of the dermatologists, were transformed into scores by adding the values assigned in the Likert scale for the items that comprised the instrument, as follows: (0), when the professional registered that they neither agreed nor disagreed with the assertion; (+2) for correct answers (equivalent to “I fully agree” or “strongly disagree” for true and false statements, respectively); and (+1), for partially correct answers (equivalent to “partially agree” or “partially disagree” for true and false, respectively). The values −2 and −1 were attributed to the incorrect or partially incorrect answers, in the opposite direction of the evaluations of correct answers. The discriminatory analysis or hypothesis test aimed to verify whether the instrument was able to discriminate between dermatologists (who should obtain higher scores) or generalists and pediatricians (who should obtain lower scores). The mean scores of the respondents were also calculated and compared, seeking association with group variables (professional training, gender, age, and personal or family history of skin cancer). Answers were considered correct when they showed partial or full agreement for the true statements, as well as partial or full disagreement for the false statements. For the comparison of the scores between groups, the Mann–Whitney *U*-test with 5% significance level was used.

#### Step 5: Temporal stability analysis (test–retest)

The temporal stability was assessed using the kappa statistic, after the instrument was re-applied to 15% of the respondents following a period of two to four weeks after the first response to the instrument. For this analysis, the dichotomous results of the answers (correct and incorrect) were considered. The following parameters were used to interpret kappa statistics: low agreement: <0.40; regular agreement: 0.41–0.60; good agreement: 0.61–0.80, and excellent agreement: >0.80.^25^ A Bland–Altman graph was plotted for validation of the test–retest, considering the total score of the instrument. The intraclass correlation coefficients were also calculated for perfect agreement for each of the instrument dimensions, considering the respective before (test) and after (retest) scores.

All statistical analyses were performed using the statistical package IBM-SPSS, v. 22.0 for Windows.

## Results

After assessment by the panel of experts in the content validation stage, the instrument, which initially included 98 items, then had 70 items. In the apparent validation stage, a consensus was reached among the group of professors to exclude a single item. During the construct validation stage, 26 items correctly answered by more than 90% of the professionals (considered to be too easy) were excluded. The exclusion of items correctly answered by less than 10% of respondents (considered to be too difficult) had been proposed, but no item reached such classification.

The main characteristics of the group of physicians who participated in the study are presented in Table 1.

**Table 1:** Characterization of the general practitioners and pediatricians who participated in the study; 2016.

Variables	(*n*)	(%)
*Sex*		
Male	64	29.5
Female	153	70.5

*Age (years)*		
<30	74	34.1
30–39	68	31.3
40–49	44	20.3
>50	31	14.3

*Specialty*		
Pediatrics	38	17.5
Clinical Medicine	103	47.5
Family and Community Medicine	76	35.0

*Main professional activity*		
Direct patient care	196	90.3
Academic career	18	8.3
Administrative function	3	1.4

*Previous or family history of skin cancer*		
Yes	39	18.0
No	178	82.0

Assessing the correlation of each item with the mean score of the complete questionnaire, two items (Q60 and Q68) that presented values lower than 0.2 for the correlation coefficient were excluded. The internal consistency analysis indicated a Cronbach's *α* of 0.780 for the final instrument with 41 items, which reflects a good level of internal consistency. For the dimension “Solar radiation and its adverse effects on the skin,” Cronbach's *α* was 0.720; for the dimension Photoprotection, this value was 0.816.

Table 2 presents the kappa statistics in the instrument reproducibility test. Over 60% of the items presented good to excellent agreement.

**Table 2:** Kappa statistics for the reproducibility of the instrument; 2016.

Kappa	Agreement rating	Items
<0.40	Low	Q43, Q45
0.41–0.60	Moderate	Q6, Q8, Q13, Q16, Q23, Q30, Q36, Q42, Q50, Q54, Q56, Q66, Q74, Q91
0.61–0.80	Good	Q12, Q18, Q20, Q24, Q28, Q33, Q35 Q53, Q55, Q57, Q58, Q65, Q73, Q78, Q82, Q85, Q92, Q94, Q95, Q96
>0.80	Excellent	Q9, Q26, Q71, Q75, Q97

For the “Solar radiation” dimension, the intraclass correlation coefficient for the maximum agreement was 0.769 (95% CI: 0.554–0.887) and for the “Photoprotection” dimension, 0.763 (95% CI: 0.578–1.874).

**Figure 2:**
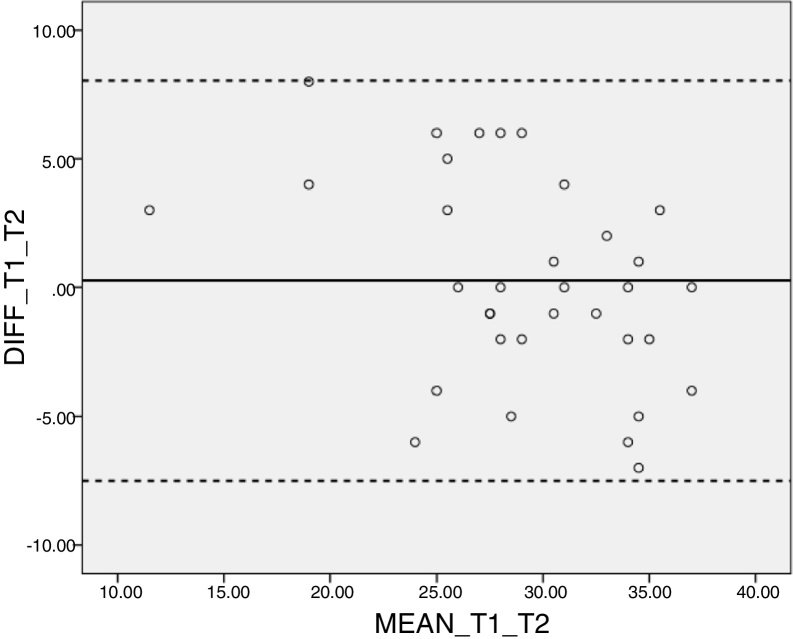
Bland–Altman plot for evaluation of the agreement of the test–retest scores.

The Bland–Altman graph indicated a satisfactory agreement between the test and retest evaluations, with little dispersion of results (Fig. 2).

The discriminatory analysis recorded a mean score of 54.1 (SD ± 8.8) points for dermatologists and 31.1 (DP ± 12.9) points for generalists and pediatricians, from a total of 82 possible points, representing a statistically significant difference (*p* < 0.001). No differences were observed among other characteristics evaluated for the group (Table 3).

**Table 3:** Comparison between the mean scores of knowledge on photoprotection among physicians; 2016.

Variable	Mean	SD	*p*-Value^a^
*Professional training 1*			<0.001
Generalists/pediatricians	30.8	12.9	
Dermatologists	54.1	8.8	

*Professional training 2*			0.175
Clinical medicine	29.2	12.9	
Family medicine	31.7	13.2	
Pediatrics	33.4	12.3	

*Sex*			0.072
Male	28.6	11.9	
Female	31.8	13.3	

*Age*			0.617
<40 years	31.4	12.6	
≥40 years	30.2	13.3	

*Family history of skin cancer*			0.296
Yes	33.1	12.9	
No	30.3	12.9	

a Mann–Whitney *U*-test.

The items with lower scores in the dimension “Solar radiation and its adverse effects on the skin” refer to the period and time of sun exposure that leads to greater damage (Q8), effects of UVA and UVB radiation (Q12 and Q18), and the classification of people into phototypes according to skin color and skin response to sunlight (Q9). In the “Photoprotection” dimension, items with lower scores addressed the combination of sunscreens with repellents (Q53), sun protection factor (Q55 and Q56), fabric types that have the greatest photoprotective effect (Q65), and use of sunscreens as the main recommendation for photoprotection (Q78). Table 4 presents the final instrument with the percentage of correct answers.

**Table 4:** Instrument for the evaluation of the knowledge of general practitioners and pediatricians about photoprotection, and percentage of correct answers; 2016.

Item (original)	Item (new)	Statement	T/F	Correct answers (%)
*Dimension 1: Solar radiation and its adverse effects on the skin*				
Q6	1	The ultraviolet index (UVI) scale is intended to simplify the disclosure of ultraviolet radiation levels to the lay public according to a table of values that ranges from 0 to 11 + .	T	57.1
Q8	2	Except in the winter, a person exposed to the sun without protection between 8 am and 5 pm can receive a dose of ultraviolet radiation superior to the recommended one.	F	36.9
Q9	3	People are classified into phototypes according to skin color and response to sunlight; the higher the phototype, the greater the incidence of skin cancer.	F	54.4
Q12	4	Sunburn is mainly caused by ultraviolet A (UVA) radiation.	F	29.5
Q13	5	Heat stroke is an exaggerated increase in body temperature after excessive exposure to sunlight.	T	87.1
Q16	6	Ultraviolet (UV) radiation causes immunosuppression, decreasing the immune response of the skin to allergenic and infectious antigens, but it also facilitates skin carcinogenesis.	T	81.1
Q18	7	Ultraviolet B (UVB) radiation is more related to carcinogenesis than ultraviolet A (UVA) radiation.	T	45.6
Q20	8	Skin cancers are associated only with chronic exposure to ultraviolet (UV) radiation.	F	65.0
Q23	9	Intense exposure to ultraviolet (UV) radiation in childhood and adolescence, resulting in severe burns, has little effect on the risk of developing melanoma throughout life.	F	73.3
Q24	10	There is a marked relationship between intermittent sunburn and the development of melanoma.	T	74.2
Q26	11	Skin cancer is the most prevalent neoplasm in several countries in the world.	T	88.5
Q28	12	Most skin cancers have low cure rates with proper treatment.	F	89.9
Q30	13	A significant portion of the sun exposure that a person receives throughout life occurs in childhood and adolescence.	T	83.9
Q33	14	The possibility of ultraviolet radiation (UV)-induced erythema is independent of skin color and skin sensitivity to the sun.	F	78.8
Q35	15	The minimum erythematosus dose (MED) refers to the smallest amount of ultraviolet (UV) radiation that is capable of causing skin erythema or slight reddening of the skin.	T	68.7
Q36	16	Childhood and adolescence are considered critical periods of vulnerability to the effects of sun exposure.	T	87.6

*Dimension 2: Photoprotection*				
Q42	17	Educational campaigns increase awareness of skin cancer, but do not always lead to behavioral changes.	T	88.9
Q43	18	Most people use only a topical photoprotector as a photoprotective measure.	T	87.1
Q45	19	Ultraviolet filters can be organic (chemical) or inorganic (physical) compounds.	T	74.2
Q50	20	In Brazil, topical photoprotectors are categorized by the Brazilian Health Surveillance Agency (ANVISA) as cosmetics.	T	59.4
Q53	21	The combination of sunscreen and insect repellents is recommended, as one product does not interfere with the other.	F	27.6
Q54	22	The sun protection factor (SPF) quantifies the protection against erythema/sunburn.	T	73.7
Q55	23	The sun protection factor (SPF) evaluates the protection against ultraviolet A (UVA) and B (UVB) radiation.	F	15.2
Q56	24	The sun protection factor (SPF) is a preventive measure against sunburn and skin cancer.	F	12.4
Q57	25	The sun protection factor (SPF) of a sunscreen generally represents less than the expected protection, since less than half of the recommended amount of sunscreen is applied.	T	65.0
Q58	26	The use of sunscreens with sun protection factor (SPF) 30 is considered adequate for the vast majority of individuals, both in the pediatric and adult populations.	T	64.5
Q65	27	Synthetic fabric (polyester, nylon) garments protect less than those made of natural fiber (cotton, silk, wool).	F	24.9
Q66	28	Densely woven fabrics (thick, closed, compact) and dark colors offer greater photoprotection.	T	52.5
Q71	29	It is recommended to consider shade as the only protection strategy.	F	83.9
Q73	30	The amount of sunscreen usually applied generally matches that recommended.	F	82.5
Q74	31	Application in insufficient quantities is the main cause for reduced effectiveness of sunscreens.	T	78.8
Q75	32	One strategy to reach the appropriate amount of protection is to apply the photoprotector in two layers (double application).	T	48.4
Q78	33	The main recommendation for photoprotection is the use of sunscreens.	F	15.7
Q82	34	The use of a topical photoprotector alone is sufficient for preventing skin cancer.	F	74.2
Q85	35	Early-life sun exposure has a crucial impact on the onset of skin cancer.	T	81.6
Q91	36	A significant portion of the ultraviolet (UV) radiation that a person receives throughout life occurs in childhood and adolescence.	T	88.9
Q92	37	Topical photoprotectors can be used since birth.	F	54.4
Q94	38	Up to 2 years of age, preference should be given to the use of organic (chemical) protectors.	F	56.2
Q95	39	Mechanical photoprotection measures, such as clothing, hats, glasses, and shade, should be stimulated in childhood and adolescence, and should prevail over the use of sunscreens.	T	61.8
Q96	40	Parents should be instructed about the shadow rule: the greater the shadow of the child projected on the floor in relation to their height, the lower the risk.	T	44.7
Q97	41	In preschool and primary school age children, photoprotection depends on the parents awareness level.	T	84.8

T/F, True or False.

## Discussion

In the present study, an instrument to assess the knowledge of general practitioners and pediatricians about photoprotection and solar radiation was elaborated and validated. The final instrument showed a satisfactory level of internal consistency, as assessed by Cronbach's alpha. The reliability of the tool can also be considered satisfactory, with a good-to-excellent agreement level for most of the evaluated items, as measured by the kappa statistic, showing adequate reproducibility. Discriminatory analysis was also able to adequately identify professionals with greater knowledge; other characteristics of the participating physicians (gender, age, specialty, and family history of skin cancer) had no influence on the means of the final score.

Although a factorial analysis of the instrument items was not conducted, internal consistency and discriminatory analysis are satisfactory measures for construct validation, according to Pasquali.^26^ For that author, factor analysis is a technique for analyzing the behavioral representation of the construct, which can also be measured by the internal consistency analysis. The discriminatory analysis demonstrated the higher scores in the group of dermatologists: the dermatologists obtained a mean score of 64% on the questionnaire, in contrast to the mean of generalists and pediatricians (37%), which represents a statistically significant difference.

The analysis of the scores of the doctors participating in the study led to the identification of knowledge gaps in the area of photoprotection. There is growing scientific evidence ratifying the deleterious effects of prolonged sun exposure on the skin, together with the recognition of the urgent need for better awareness of the general population regarding healthier sun exposure habits.^2^, ^4^, ^8^, ^15^, ^16^ However, healthcare professionals do not appear to be committed to this goal. Photoprotection education is still a dermatologists task, although it should be part of the primary care strategies, considering that skin cancer is the most prevalent neoplasm in Brazil and worldwide, and that the disease has been increasingly understood as a public health issue.^1^, ^2^, ^4^, ^14^

The American Academy of Pediatrics, in conjunction with the US Centers for Disease Control, has developed a guide for primary and secondary skin cancer prevention.^12^ However, shortcomings in the understanding of sun-protective behaviors and education in skin cancer in general have been observed.^12^ Weinstein et al. assessed the knowledge and attitudes of over 200 parents in pediatric and dermatological clinics regarding photoprotection, and observed that their sources of information on the subject came mainly from the media (television, magazines, radio), but that they wanted to get information from their primary care physicians.^12^, ^27^ That is: the recommendations exist and physicians are considered a safe source of information by the population, and should be included in strategic photoprotection education planning.^17^ While there are questionnaires to evaluate sun exposure and its association with skin cancer, until now there was no validated questionnaire to measure the knowledge of physicians about photoprotection. Knowledge is an important parameter and represents the first step in any program that aims for long-term results.

In the present study, the exclusion of items from the instrument because they were correctly answered by more than 90% of professionals (considered to be too easy) indicates that participants demonstrated an understanding of the relationship between excessive sun exposure and skin cancer and the importance of primary prevention in efforts to combat melanoma, showing that the group recognizes the relevance of the topic. The participants also demonstrated knowledge of the concept of photoprotection, the available photoprotection measures, the need to approach the subject as a set of interventions, the need to reapply topical photoprotectors, and the importance of offering a differentiated approach to the pediatric population (items that were excluded from the final instrument). Therefore, some general and superficial concepts apparently are in the public domain, contrasting with the lack of technical information demonstrated in the other items, which represent necessary information in the practice of primary care professionals.

On the other hand, less than 30% of the participants demonstrated knowledge about the higher relevance of UVB over UVA in sunburn and skin carcinogenesis, exposing a clear conceptual flaw in a topic of absolute relevance. Conversely, over 80% of the respondents agree that childhood and adolescence are critical periods in relation to sun exposure, corroborating the need for instruction of healthcare professionals, reinforcing the imperative recommendation that guidelines on safe sun exposure are implemented early enough to allow changes in attitudes and behaviors throughout life.^12^

Approximately 85% of the participants considered the use of sunscreens as the main photoprotective strategy, which goes against the recommendation of the main guidelines on the subject. Although they are considered excellent methods of photoprotection, sunscreens should be part of a range of measures that include changes in lifestyle, active pursuit of shade, and use of protective clothing, wide brim hats, and sunglasses.^28^ For the American Academy of Dermatology, the use of topical photoprotectors is considered the third line in photoprotective strategy, after clothing and shade.^6^, ^8^

Less than 20% of the respondents demonstrated knowledge about the definition and interpretation of the solar protection factor (SPF), which also indicates an important knowledge gap, since SPF is considered the main information about the effectiveness of a sunscreen.^8^, ^22^ The results suggest that the subject is probably little discussed in medical schools, being restricted to specialists. Another question is whether the lack of knowledge about sunscreens could be related to the fact that these products are classified as cosmetics in Brazil and in most countries, which could give rise to the misinterpretation that their use is optional, shifting the focus from healthcare promotion and reducing its relevance in the prevention of skin neoplasias. Also regarding topical photoprotection, less than 30% demonstrated knowledge about the interaction between sunscreens and insect repellents, which is a cause for concern in light of the recent epidemics of dengue fever, Zika, chikungunya, and yellow fever, which led to frequent repellent use by a considerable percentage of the population.

Regarding the use of clothing as a mechanical photoprotection strategy, less than 30% of the participants demonstrated knowledge about which fabrics have the greatest protection power, which is worrying, since this has been selected as the main measure to be emphasized in the educational efforts for the population regarding conscious exposure to UV radiation.^14^ The use of clothing is an easily available and effective approach to protecting the body against the harmful effects of UV radiation. Stiffer and thicker fabrics, more firmly interwoven and darker in color, have a greater protective capacity; polyester is the material with the greatest capacity for absorbing UV light and cotton is the material with the lowest capacity.^28^, ^29^

Less than half of the participants were familiar with the shadow rule, which determines that the greater the length of a shadow, the lower the risk of sun exposure, and *vice versa*.^10^ This is an important theoretical gap, since childhood appears to be the ideal time to intervene in terms of protective behaviors in the sun and the shadow rule is a simple and effective strategy, recommended in the educational approach of children.^8^

The limitations of this study include: small sample size (despite a good item/respondent relation), priority participation of female and young professionals, and regional application (in a medium-sized city in northern Minas Gerais, Brazil). The application of previously acquired knowledge is another important aspect that was not addressed in this study – a combination of knowledge assessment and measurement of the prescribed prevention methods would provide a more comprehensive picture of the situation. It is not possible to evaluate the behavior of health professionals from an isolated evaluation of knowledge, but knowledge is fundamental for professionals to adopt appropriate behaviors in their practice, guiding their choices and attitudes.

## Conclusion

This study successfully completed the elaboration of the instrument “Knowledge of general practitioners and pediatricians about photoprotection,” which was validated with good internal consistency and good reproducibility, as measured by the test-retest. It was possible to identify important knowledge gaps among professionals participating in the study. The final instrument should be considered as a valuable tool in identifying knowledge gaps of pediatricians and general practitioners on photoprotection, and may serve as a basis for the development of training and educational strategies for these physicians in different regions.

## Author's contribution

Fernanda Mendes Araújo: Statistical analysis; approval of the final version of the manuscript; conception and planning of the study; elaboration and writing of the manuscript; obtaining, analyzing and interpreting the data; effective participation in research orientation; intellectual participation in propaedeutic and/or therapeutic conduct of the cases studied; critical review of the literature; critical review of the manuscript.

Julliana Andrade do Carmo: Approval of the final version of the manuscript; conception and planning of the study; obtaining, analyzing and interpreting the data; critical review of the manuscript.

Letícia Diniz Cunha: Approval of the final version of the manuscript; conception and planning of the study; obtaining, analyzing and interpreting the data; critical review of the manuscript.

Igor Monteiro Lima Martins: Approval of the final version of the manuscript; obtaining, analyzing and interpreting the data; critical review of the manuscript.

Airton dos Santos Gon: Approval of the final version of the manuscript; conception and planning of the study; elaboration and writing of the manuscript; effective participation in research orientation; intellectual participation in propaedeutic and/or therapeutic conduct of the cases studied; critical review of the literature.

Antônio Prates Caldeira: Statistical analysis; approval of the final version of the manuscript; conception and planning of the study; elaboration and writing of the manuscript; obtaining, analyzing and interpreting the data; effective participation in research orientation; intellectual participation in propaedeutic and/or therapeutic conduct of the cases studied; critical review of the literature; critical review of the manuscript.

## Financial support

None declared.

## Conflicts of interest

None declared.
